# Proteomic Analysis Provides New Insights in Phosphorus Homeostasis Subjected to Pi (Inorganic Phosphate) Starvation in Tomato Plants (*Solanum lycopersicum* L.)

**DOI:** 10.1371/journal.pone.0134103

**Published:** 2015-07-29

**Authors:** Sowbiya Muneer, Byoung Ryong Jeong

**Affiliations:** 1 Division of Applied Life Science (BK21 Plus), Graduate School, Gyeongsang National University, Jinju, 660–701, South Korea; 2 Institute of Agriculture & Life Science, Gyeongsang National University, Jinju, 660–701, South Korea; 3 Research Institute of Life Science, Gyeongsang National University, Jinju, 660–701, South Korea; Universidade Federal de Vicosa, BRAZIL

## Abstract

Phosphorus is a major nutrient acquired by plants via high-affinity inorganic phosphate (Pi) transporters. To determine the adaptation and homeostasis strategy to Pi starvation, we compared the proteome analysis of tomato leaves that were treated with and without Pi (as KH_2_PO_4_) for 10 days. Among 600 reproducible proteins on 2-DE gels 46 of them were differentially expressed. These proteins were involved in major metabolic pathways, including photosynthesis, transcriptional/translational regulations, carbohydrate/energy metabolism, protein synthesis, defense response, and other secondary metabolism. The results also showed that the reduction in photosynthetic pigments lowered P content under –Pi treatments. Furthermore, high-affinity Pi transporters (*lePT1* and *lePT2*) expressed in higher amounts under –Pi treatments. Also, the accumulation of Pi transporters was observed highly in the epidermis and palisade parenchyma under +Pi treatments compared to –Pi treatments. Our data suggested that tomato plants developed reactive oxygen species (ROS) scavenging mechanisms to cope with low Pi content, including the up-regulation of proteins mostly involved in important metabolic pathways. Moreover, Pi-starved tomato plants increased their internal Pi utilization efficiency by increasing the Pi transporter genes and their rational localization. These results thus provide imperative information about how tomato plants respond to Pi starvation and its homeostasis.

## Introduction

Phosphorus (P) is an essential macronutrient necessary for plant growth and development [1–3]. Pi (inorganic phosphate) is the key source of phosphorus uptake by plants transported from a plasma membrane of epidermal and cortical cells [4]. Phosphorus is transported through other cell membranes within the plants via Pi transporters [5]. However, Pi concentration is extremely low in many soils due to the poor mobility of phosphate [4]. To cope with Pi limitation, plants have developed an organized mechanism to maintain homeostasis such as the acquisition of Pi from soil and remobilization, as well as improving cellular metabolism of plants for Pi uptake [6]. These organized mechanisms under Pi starvation have resulted a reprogramming of plant metabolism by studying gene expressions, DNA microarrays [7–9], proteomics [10–13], and metabolite profiling [10–13]. Meanwhile, relatively little information is documented about proteome network contributing to homeostasis in Pi starvation. Proteomics is an ideal tool for characterizing the protein profile of plants because it not only provides an overview of proteins but also assists in the detection of signal transduction pathways and protein function [14–16].

Several physiological studies have validated that plants respond to Pi starvation in different ways, including improving Pi acquisition and internal Pi remobilization. The mechanisms fundamental for these processes include the production of phosphate transporters [5] and altered root/leaf morphology [17, 5]. The physiological responses associate with Pi starvation have been linked to differences in gene expression profiles [18–19]. The expression of genes involved in photosynthesis such as PSI, PSII, ribulose-1, 5-bisphosphate carboxylase/oxygenase (RuBisCO), and pigment proteins such as Chlorophyll a/b binding proteins were inhibited by Pi starvation. The abundance of other transcripts involved in Pi transport such as PHT1, PHT2, PHT3, and PHT4 encoding proteins involved in Pi acquisition across the plasma membrane, chloroplast, and mitochondria [20–21] were up-regulated under Pi starvation. The other Pi transcripts, including members of the PHO1 family, also up-regulate during the Pi starvation for Pi acquisition [22].

Tomato is the widest range of growing crops in all countries for its fruit consumption and is considered as a model plant for the investigation of mineral deficiencies and other abiotic stresses [23–24]. In this work, we determined the adaptation and homeostatic strategy to Pi starvation. To follow our hypothesis we measured physiological changes in plants such as phenotypic differences, pigment analysis, and P content. For proteomic analysis we followed second dimensional gel electrophoresis (2-DE) and mass spectrometry to identify proteins involved in the tolerance and homeostasis of Pi starvation. Furthermore, we also studied gene expression analysis of phosphorus transporters (*lePT1* and *lePT2*) and their localizations under Pi starvation.

## Materials and Methods

### Plant materials and growth conditions

Seeds of tomato (*Solanum lycopersicum* L.) var. ‘Golden tomato’ obtained from Jeil Seed Company (Jeungpyeong-gun, Korea) were disinfected using 1% sodium hypochlorite (NaClO) followed by ten washing with 100 ml distilled water. The seeds were germinated on a square plug tray containing commercial Tosilee medium (Tosilee medium, Shinan Precision Co., Jinju, Korea) for one week. After germination, seedlings with uniform size were divided into two sets receiving hydroponic nutrient solution containing (mM for the macro elements): 2 KNO_3_; 1 Ca(NO_3_)_2_.4H_2_O; 2 MgSO_4_.7H_2_O; 1 NH_4_NO_3_; (mM microelements) 14 H_3_BO_3_; 5.0 MnSO_4_∙H_2_O, 3.0 ZnSO_4_∙7H_2_O; 0.7 CuSO_4_∙5H_2_O; 0.7 (NH_4_) 6MO_7_O_2_4; 0.1 COCl_2_ and 1 M KH_2_PO_4_ 5; (Fe-stock) 8 μM Fe(III)-EDTA. The nutrient solution was supplied to Tosilee medium every 3 days and pH (5.8) was continuously maintained in blocks. For complete Pi starvation, the Pi was completely removed from a hydroponic solution by avoiding the addition of KH_2_PO_4_. The plants were grown in plant growth chamber under fluorescent light at 100 μmol m^-2^ s^-1^ at the canopy height for 16 h day^-1^ at a relative temperature of 25°C. After 10 days of treatment, leaves and roots were excised from the main plant and immediately frozen in liquid N_2_ and stored in a deep-freezer (-80°C) for further analysis. For chemical analysis, the plants were oven dried at 70°C for 48 h and used for the experiments.

### Physiochemical characteristics

#### Measurement of P content and pigment analysis

For the determination of P content, 1 g of oven-dried leaf samples was digested with 50% perchloric acid and concentrated H_2_SO_4_ at 100–300°C for 5 h. The digested samples were then filtered with whatman filter paper number 6 and was diluted to a final volume of 50 ml by adding double distilled water. The elemental content was determined by inductively coupled plasma optical emission spectrometry, (ICP-OES, Thermo Elemental—IRIS Advantage, USA).

Total chlorophyll and carotenoid content were determined by dimethyl sulfoxide (DMSO) as earlier described by Hiscox and Israclstam [25]. Fresh leaves were collected in a glass vial to which 5 ml of DMSO were added and were kept in an oven at 65°C for complete extraction of pigments. The extracts were read by a UV-Vis spectrophotometer at 480, 645, 520 and 663 nm. The pigment concentrations in mg fresh samples were calculated using the formulae given by Arnon [26].

#### H_2_O_2_ and O_2_^-1^ localization

To visualize H_2_O_2_ localization, leaves from all the treatments were immersed in a 1% solution of 3,3'-diaminobenzidine (DAB) (Sigma-Aldrich, St. Louis, MO, USA) in Tris-HCl buffer (pH 6.5), vacuum-infiltrated for 5 min placed in closed vacuum jar attached with suction pump to apply and release vacuum. After vacuum infiltration leaves were incubated at room temperature (25°C) for 2–3 h in the absence of light. Leaves were illuminated until the appearance of brown spots characteristic of the reaction of DAB (Sigma-Aldrich, St. Louis, MO, USA) with H_2_O_2_ (hydrogen peroxide). Leaves were bleached by immersing in boiling ethanol to visualize the brown spots and were photographed with a digital camera (Nikon, Japan) at a default setting of 600 dpi.

For the visualization of O_2_^−1^, leaves were immersed in a 0.1% solution of nitro blue tetrazolium (NBT) (Sigma-Aldrich, St. Louis, MO, USA) in K-phosphate buffer (pH 6.4), containing 10 mM Na-azide (Sigma-Aldrich, St. Louis, MO, USA), and were vacuum-infiltrated for 5 min placed in closed vacuum jar attached with suction pump to apply and release vacuum. After vacuum infiltration leaves were illuminated until the appearance of dark blue spots (characteristic of blue formazan precipitate). After bleaching in boiling ethanol, the leaf samples were photographed as described above.

### Proteomic analysis

#### Protein profile by first dimension SDS-PAGE

The samples of leaf were immediately harvested after a plant being uprooted, weighed and frozen in liquid nitrogen followed by grinding in chilled pestle and mortar to a fine powder. This powder was extracted with 40 mM (w/v) Tris-HCl, pH 7.5, 2 mM (w/v) EDTA, 0.07% (w/v) β-mercaptoethanol, 2% (w/v) PVP and 1% (v/v) Triton X-100. The extract was centrifuged at 13,000 rpm for 10 min at 4°C. The supernatant was mixed with 6-X protein-dye containing 240 mM Tris-HCl (pH 6.8), 40% glycerol, 8% SDS, 0.04% bromophenol blue and 5% beta-mercaptoethanol. The samples containing 40 μg proteins were loaded on 12.5% polyacrylamide gel on PROTEAN II (Bio-Rad, Hercules, CA, USA). The protein concentration was determined by Bradford method using BSA (bovine serum albumin) as a standard curve. After electrophoresis, the gels were stained with a commercial available silver stain according to manufacturer’s instructions (Bio-Rad, Hercules, CA, USA).

#### Protein sample preparation for 2-DE

Leaves from tomato plants were harvested and homogenized in liquid nitrogen in precooled pestle and mortar. The proteins were extracted in commercially available protein extraction buffer kit (Bio-Rad, Hercules, CA, USA) according to manufacturer’s instructions. About 1 ml of extraction buffer containing 8M urea, 4% CHAPS, 40 mM Tris, 0.2% (w/v) bio-lyte (*pI* 3–10) were added to 100 mg of powdered frozen samples. The samples were vortexed and placed on ice, then sonicated with an ultrasonic probe to disrupt the cells and fragments of the genomic DNA. The sonicated samples were centrifuged at 13, 000 rpm in a microcentrifuge for 20 min at 4°C to pellet down the cell debris. The resulting supernatant were transferred to a clean e-tubes and extracted protein samples in the form of supernatant were quantified by Bradford using BSA (bovine serum albumin) as a standard curve.

#### Two-dimensional gel electrophoresis (2-DE) and silver staining

For isoelectric focusing (IEF), the Multiphor II system (GE Healthcare) and IPG strip (pH 4–7, nonlinear, 11 cm, GE Healthcare) were used according to manufacturer’s instructions with minor modifications. To minimize experimental errors, extracted protein samples from three biological replicates were subjected to focussing at the same time. The dry IPG strips were rehydrated for 12 h in 250 μl rehydration buffer (Bio-Rad, Hercules, CA, USA) containing 100 μg of protein. Focusing was performed at 20°C at a current limit of 50 μA per IPG strip at 20°C in four steps: 200 V for 0:01 (h:min), 3500 V for 1:00 (h:min), 3500 V for 1:30 (h:min), and a final step 3500 V for 1–2 h until a final current of 10,000 Vh was reached. The gel strips were then equilibrated by incubating on a shaker in 2–3 ml of an equilibration buffer 1 for 30 min [8M urea, 2% SDS, 50 mM Tris-HCl (pH 8.8) 20% (v/v) glycerol, 1% DTT] followed by 2–3 ml of an equilibration buffer 2 for 30 min [same content as equilibration buffer 1 except DTT was replaced by 2.5% iodoacetamide]. The second dimension was performed on 12.5% (w/v) SDS-polyacrylamide gels on PROTEAN II (Bio-Rad, Hercules, CA, USA) with a constant voltage of 70–100 V for 4–5 h until the run was complete. For molecular weight of proteins, commercial pre-stained molecular marker (Intron Biotechnology, Seongnam-City, South Korea) were run on one side of the SDS-PAGE gels. Protein spots were visualized by staining 2D gels with silver stain. The stained gels were scanned using a high resolution scanner (EPSON) and gel images were analyzed using PD-Quest basic software (Bio-Rad, Hercules, CA, USA). The 2D gels were stored in 1% acetic acid until further analysis.

#### Image and data analysis

In each treatment, three independent biological replicate plants were taken. Gel images were taken under constant settings by a photo imager. PDQuest version 7.2.0 (Bio-Rad, Hercules, CA, USA) was used to assemble a first level match set (master image) from three replicate 2-DE gels. Further, protein quantification was analyzed as described previously [15].

#### Protein in gel digestion

The differential protein spots were excised manually from the 2D gels with the help of a clean razor blade and were chopped into small pieces. The excised spots were transferred to 0.5 ml clean microfuge tubes. The gel pieces were destained with freshly-prepared 30 μl of a 1:1 (v/v) mixture of the two destained reagents K_3_[Fe(CN)_6_] (potassium ferricyanide) and Na_2_S_2_O_3_ (sodium thiosulphate pentahydrate) by incubating for 30 min at room temperature (25°C) with gentle agitation. The destaining solution was removed and gel particles were washed with distilled water and 50 mM NH_4_HCO_3_/ACN (v/v) (ammonium bicarbonate/acetonitrile) for 15 min (1:1). The gel particles were then covered again with ACN (acetonitrile) for 2–5 min and were dried in a vacuum centrifuge. After drying, the gel particles were rehydrated in 10 mM dithiothreitol /50 mM NH_4_HCO_3_ (ammonium bicarbonate) (1:1) by incubating at 56°C for 45 min. The e-tubes containing gel particles were cooled to room temperature (25°C) in dark conditions and a rehydrated solution was removed. The gel particles were again washed with 50 mM NH_4_HCO_3_ (ammonium bicarbonate) and ACN (acetonitrile) (1:1) with one or two changes for 15 min per change. The gel particles were covered with ACN (acetonitrile) to shrink the gel pieces and then dried in a vacuum centrifuge. After washing steps, the gel particles were treated with freshly prepared 5 ng of trypsin (Sigma-Aldrich, St. Louis, MO, USA) prepared in 1 M HCl to cover the gel and was incubated overnight at 37°C to keep gel. After overnight incubation the microfuge tubes containing gel particles were spun down and resulting supernatants (peptide mixtures) were collected in new microfuge tubes. The resulting peptides were vacuum dried and dried peptides were dissolved in a 3–5 μl of sample solution containing 50% ACN (acetonitrile) and 0.1% TFA (trifluoroacetic acid). The solutions were stored at -20°C until further use.

#### Protein identification using MALDI-TOF MS and MS/MS analysis

The digested peptide solution was spotted onto the MALDI-TOF MS target plate with a pipette. MALDI-MS analysis was performed with a Voyager DE-STR mass spectrometer (Applied Biosystems, Framingham, MA, USA). A two-point internal standard [des-Arg1-Bradykinin (m/z 904.4681) and neurotensin (m/z 1672.9175)] was used for calibration. The software Data Explorer (Perspective Biosystems, Inc., USA; v5.0) was used to view and process data files. The peptide mass fingerprint (PMFs) obtained from each digested protein were compared with PMFs in the non-redundant National Center for Biotechnology Information database (NCBInr, 2011/02/01, entries from all green plants) using the MASCOT database (http://www.matrixscience.com). An ABI 4800 Plus TOF–TOF Mass Spectrometer (Applied Biosystems, Framingham, MA, USA) was employed for MS and MS/MS analyses of the peptides. The instrument was set at 200 Hz ND: 355 nm YAG laser operations. Signal/noise ratios > 25 (1:1) and the ten with higher intense ions were used to following MS/MS analysis in 1 kV mode, 1000–1250 consecutive laser exposure. The MS and MS/MS spectra data were analyzed using NCBI and Protein Pilot V.3.0 database software (with the MASCOT V.2.3.02 database search engine) at 50 ppm of mass tolerance. Oxidation of methionines and carbamidomethylation of cysteines were allowed for the MS/MS spectra search in the databases. Individual peptide ion scores were searched using a statistically significant threshold value of *p* = 0.05.

#### Protein functional classifications

The identified proteins were classified into different categories of biological processes in which they are involved according to gene ontology (http://www.geneontology.org/).

### Gene analysis

#### RNA isolation, cDNA preparation and RT-PCR

Isolation of RNA from the leaves (same leaves used for protein analysis) was performed using an RNA isolation kit according to the manufacturer’s instructions (Promega, Madison, WI, USA). One μg of DNAase-treated RNA was reverse transcribed using a reverse transcriptase kit (Promega, Madison, WI, USA) to synthesize first-strand cDNA. Quantitative Real-time PCR was performed with a Rotor-Gene Q 2plex HRM Platform (Rotor-Gene Q 2plex HRM Platform), using SYBR green as a reference dye provide by Qiagen qPCR kit (QIAGEN OneStep RT-PCR Kit, Westburg, Netherland) for 5 min at 95°C, followed by 25 cycles consisting of 20 sec at 95°C, 30 sec at 57°C and 30 sec at 72°C, then 10 min at 72°C. All quantifications were normalized to actin. The RT-PCR reactions were performed using three independent RNA preparations from independently grown plants and three replicates for qPCR. The gene specific primers used in this study are as follows: *lePT1* (accession number: AF022873.1) F-5’-ATAAAAATGCAAAATAATCC-3’; R-5’-AGCCACCCGAAGAACAACTG-3’; *lePT2* (accession number: AF022874.1) F-5’-AGAAAGTGCACAATTTTTTG-3’; R-5’-GGTGTACTACCAAAGGAGAG-3’; *leActin* (accession no. U60482) F-5’-CTGCCATGTATGTTGCCATC-3’; R-5’-GGCTGTGGTGGTGAAAGAGT-3’.

#### *In situ* localization of tomato phosphate transporters transcripts

Leaves of tomato plants grown in Tosilee medium under +Pi and–Pi conditions were harvested and transversely cut into 1–3 mm^2^ pieces, and fixed in a solution containing 3.7% (v/v) formaldehyde prepared in phosphate buffer (pH 7.2) for 2 h. The fixed samples was dehydrated in ethanol dilution series viz., 35, 50, 70, 95, and 100% 15 min each and samples were cut into thin transverse sections using a microtome. The samples (transverse sections) were placed on a glass slide and were stained with antisense probes representing *lePT1* and *lePT2*, covered with a cover slip. The resulting samples were observed under a light microscope (Nikon Eclipse Ci-S/Ci-L, Japan) under 10X magnification.

### Statistical analysis

For physiological parameters, a uniform block design was used with three replicates for two treatments and one sampling date. An individual block containing one plant represented a replicate. The Tukey’s studentized range test was employed to compare the means of separate replicates. The conclusions are predicted on differences between the means, with a significance level set at *P* < 0.05. For proteomic data, the coefficient of variation was computed for the matched spot quantities in each set of experiments with the replicate groups. All results were expressed as the Mean±SE. Differences between proteins spots in all treatments were determined using one-way analysis of variance followed by a Student’s t-test with *p* < 0.05 as the limit of significance.

## Results

### Phenotypic changes and the physiological response in tomato leaves to Pi starvation

**Fig 1:**
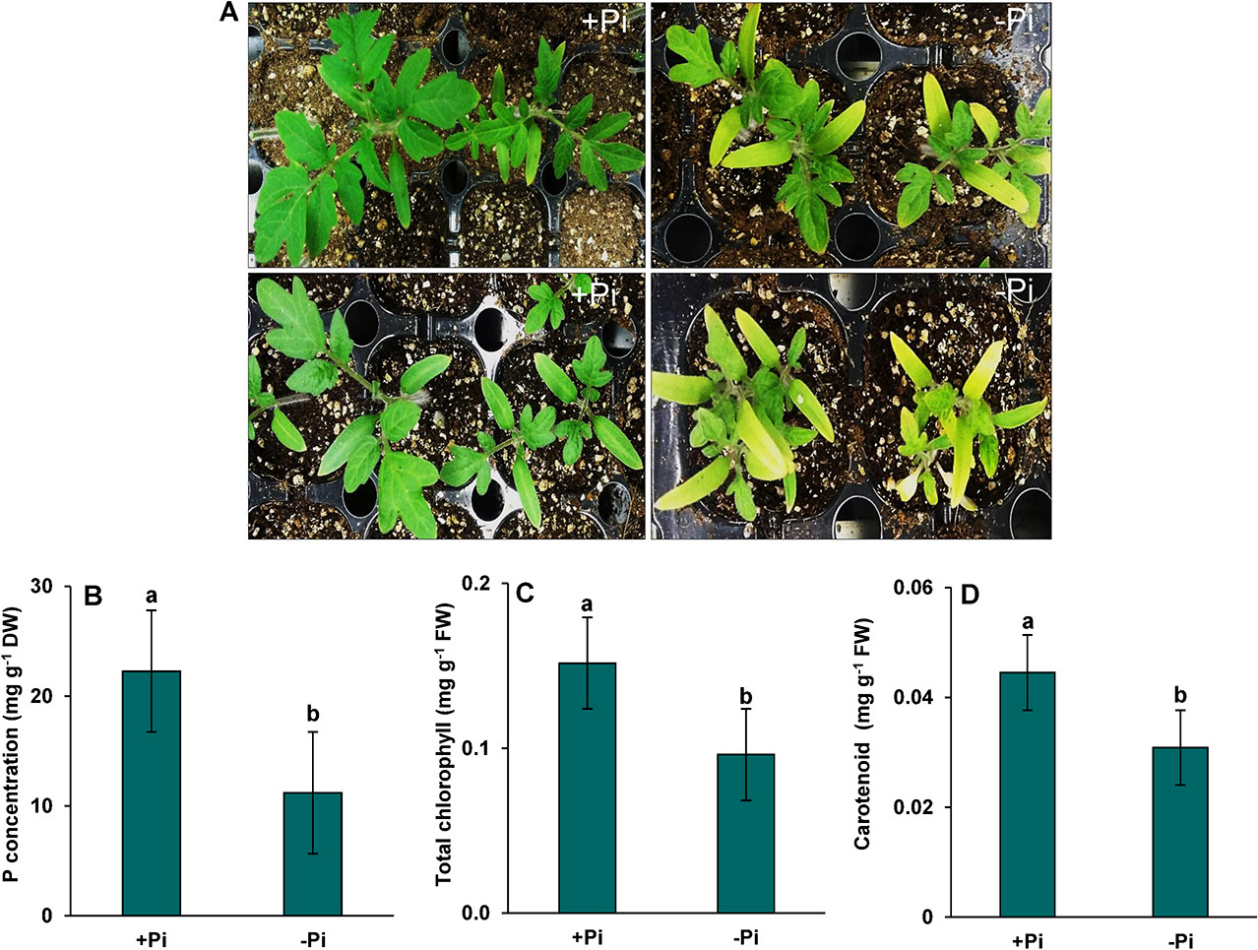
Phenotypic and physiological indices in leaves of tomato (*Solanum lycopersicum* L.) to Pi starvation. One week after germination tomato plants were supplied with sufficient Pi (1 M KH_2_PO_4_) or deficient Pi (0 M KH_2_PO_4_) for 10 days. (A) Phenotypic differences (B) Pi concentration (C) total chlorophyll and (D) carotenoid content. Vertical bars indicate Mean±SE of the means for n = 3. Means denoted by the different letter are significantly different at P≤0.05 according to the Tukey’s studentized range test.

After 10 days of Pi starvation, the tomato leaves displayed apparent Pi deficiency symptoms, including yellowing of leaves (chlorotic lesions) (Fig 1A) and a significant decline in P content (Fig 1B). Pi starvation significantly decreased photosynthetic pigments (total chlorophyll and carotenoid content) to 70% compared to Pi sufficient plants (Fig 1C and 1D).

**Fig 2:**
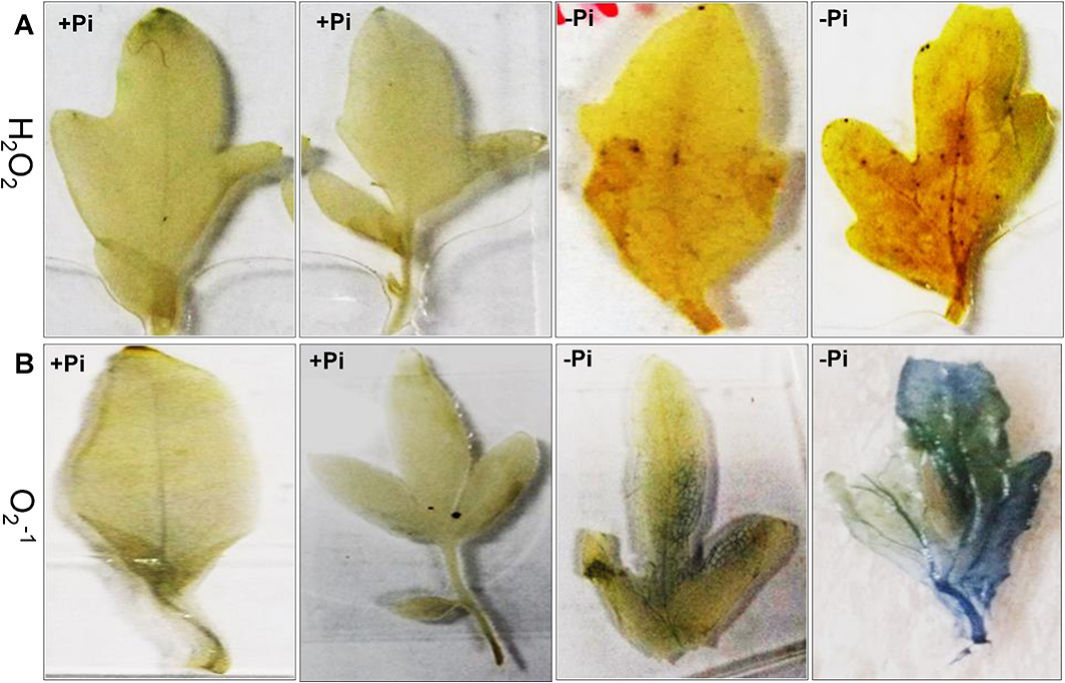
Histochemical localization in leaves of tomato (*Solanum lycopersicum* L.) to Pi starvation. One week after germination tomato plants were supplied with sufficient Pi (1 M KH_2_PO_4_) or deficient Pi (0 M KH_2_PO_4_) for 10 days. (A) H_2_O_2_ by DAB staining (B) O_2_^-1^ by NBT staining, dark brownish parts indicate localization of H_2_O_2_ and blue parts indicate localization of O_2_^-1^.

For the production of ROS, two important oxidative stress markers were visualized *in situ* in the form of H_2_O_2_ and O_2_^-1^ by histochemical methods. For visualizing H_2_O_2_ accumulation, the DAB reaction based on the formation of brownish parts was used. Pi-starved leaves displayed brownish staining compared to +Pi treatments (Fig 2A). The O_2_^-1^ was studied by a reaction with nitro-blue tetrazolium (NBT), giving rise to dark-blue spots of blue formazan. In Pi starved leaves, dark blue spotted areas were prevalent (Fig 2B) whereas no blue spotted area was observed in Pi-sufficient leaves.

### Proteome changes in tomato leaves to Pi starvation

**Fig 3:**
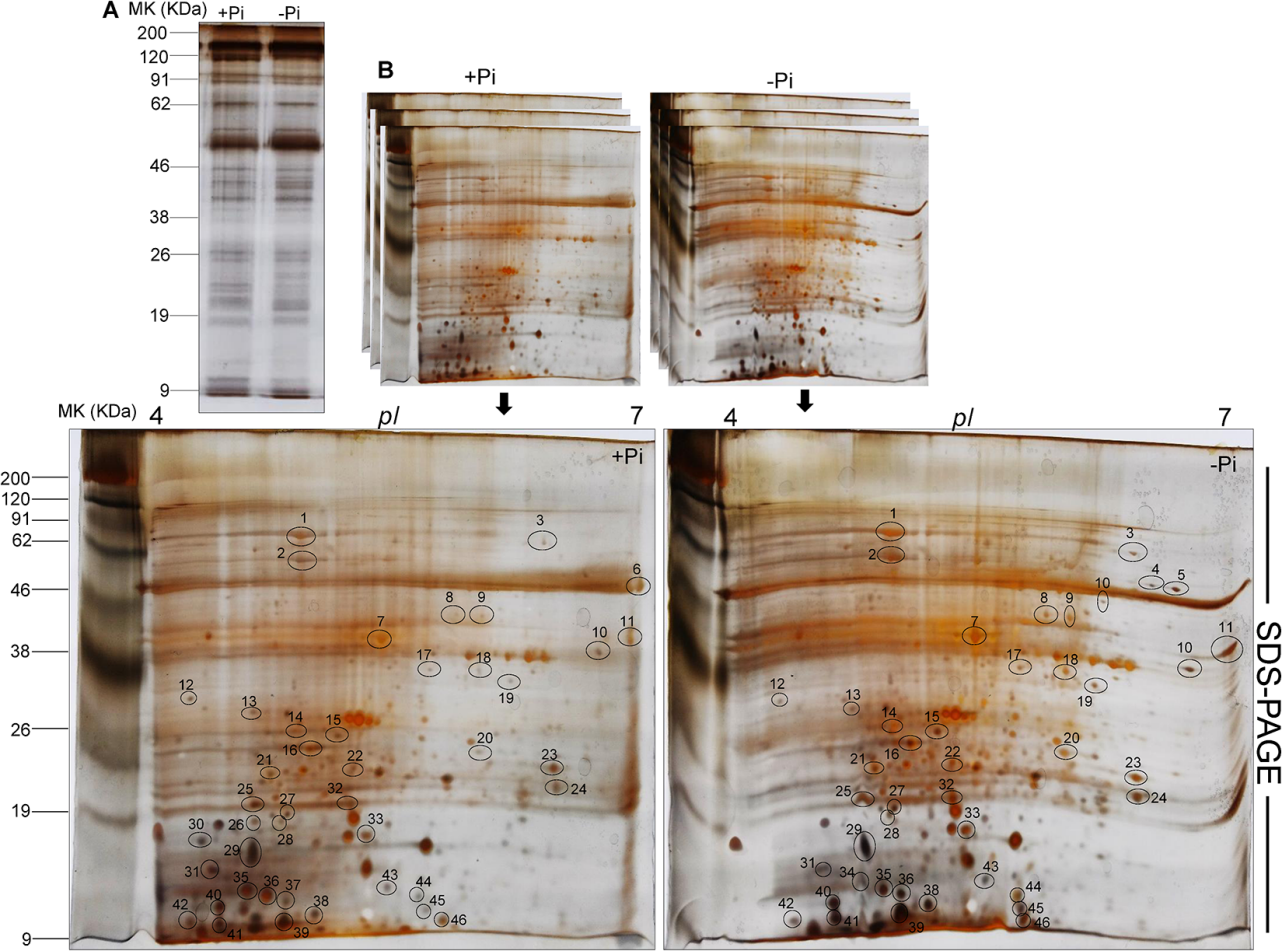
Protein profile in leaves of tomato (*Solanum lycopersicum* L.) to Pi starvation. One week after germination tomato plants were supplied with sufficient Pi (1 M KH_2_PO_4_) or deficient Pi (0 M KH_2_PO_4_) for 10 days. (A) Comparative analysis of protein profile by first dimension SDS-PAGE for analysis of enrichment of proteins (B) Comparison of 2-DE gel maps of proteins. The proteins were extracted using a commercial available kit and 100 μg protein samples were separated by isoelectric focusing (IEF) using 11 cm pH 4–7 IPG strips. The focused strips were placed on a 12% polyacrylamide gel for second-dimensional separation and stained with silver stain. The gel image analysis was carried out using PDQuest software. The encircled protein spots marked with numbers were differentially expressed. All differentially protein spots were identified by MALDI-TOF MS listed in Table 1.

**Fig 4:**
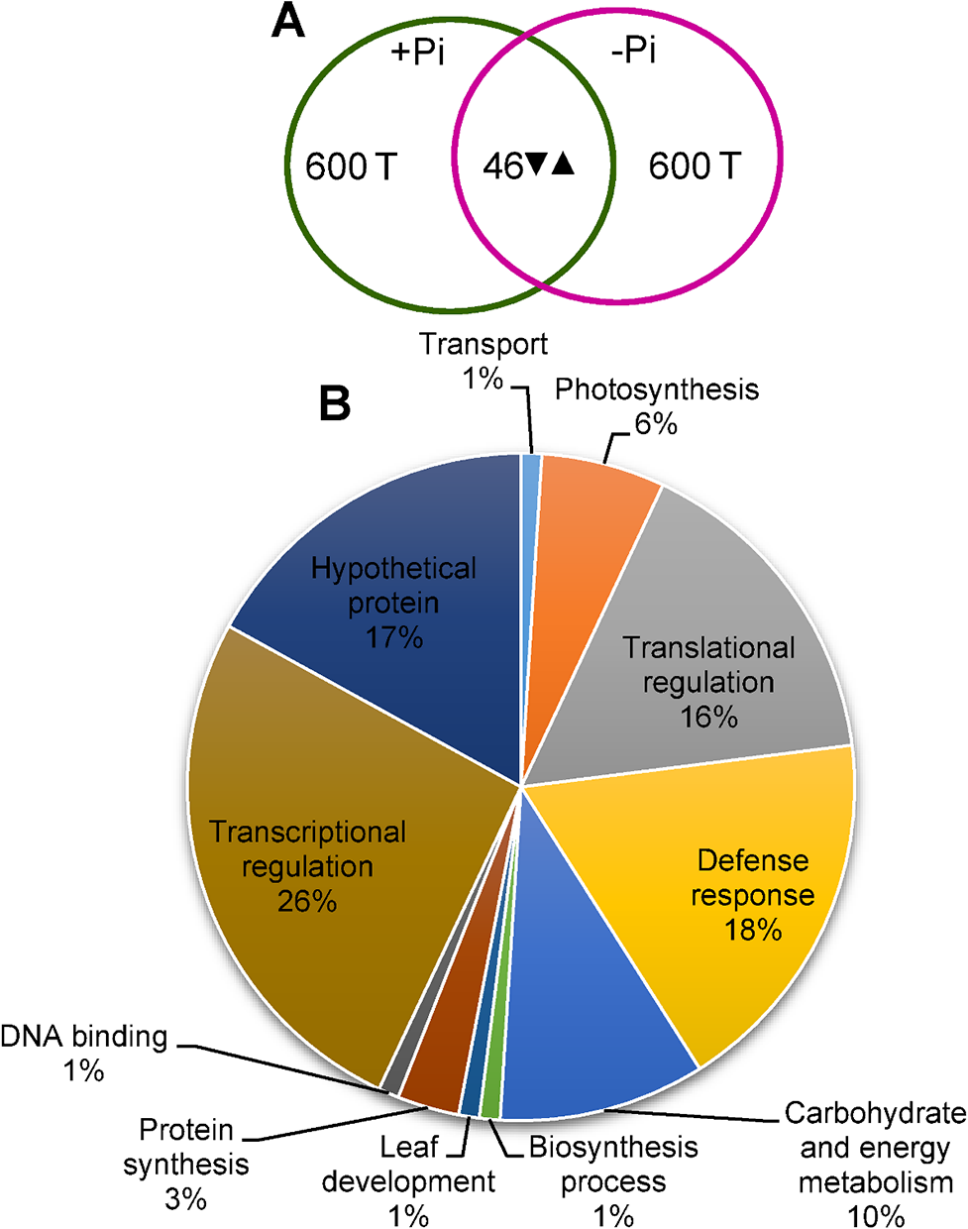
Comparative analysis of protein profile in leaves of tomato (*Solanum lycopersicum* L.) to Pi starvation. One week after germination tomato plants were supplied with sufficient Pi (1 M KH_2_PO_4_) or deficient Pi (0 M KH_2_PO_4_) for 10 days. (A) Venn diagram, the numbers in Venn diagram correspond to the protein spots present in 2-DE patterns. *Upward* and *downward* arrows denote increased or decreased protein expression under combined treatments. (B) Functional classification of identified proteins, the proteins identified were classified based on their putative biological functions according to gene ontology (www.geneontology.com).

The relative total protein profile were first analyzed by first dimension sodium dodecyl polyacrylamide gel electrophoresis (SDS-PAGE) to check the enrichment of proteins (Fig 3A). The detailed comparative 2-DE images were analyzed by PD-Quest software (Bio-Rad, Hercules, CA, USA) in the leaves of tomato subjected to Pi starvation (Fig 3B). About 600 protein spots were detected on each 2-DE gels (Fig 4A). Among 600 protein spots, 46 protein spots were differentially expressed (Figs 3B and 4A) between +Pi and–Pi treatments. It was observed that most of the protein spots in–Pi were up-regulated compared to +Pi treatments. A possible number of proteins in +Pi was also observed to be absent compared to–Pi 2-DE gels. The results indicate that Pi-starved treatments resulted in major proteomic changes in the tomato leaves.

### Identification and classification of proteins involved in tomato leaf to Pi starvation

The differentially expressed proteins were isolated and analyzed using MALDI-TOF MS. All 46 differentially expressed proteins were successfully identified (Table 1). Most of the proteins identified by a mass spectrometer had functional annotations in the universal protein data bank, whereas only a few of proteins were unknown or uncharacterized proteins having no functional annotations. The seven up-regulated protein spots in–Pi 2-DE gels were related to defense response proteins and were identified as Non-specific lipid-transfer protein 1(spot 7 and spot 9), Probable WRKY transcription factor 13 (spot 8), Extra-large G protein 3, partial (spot 15), Asymmetric leaves1 and rough sheath, putative (spot 28), and Extra-large G protein 3 partial (spot 33). The other three proteins up-regulated in–Pi 2-DE gels were related to photosynthesis and were identified as Mitochondrial-like, partial (spot 3), Putative cyclin-L2 (spot 36), and Ribosomal protein S9 (chloroplast) (spot 46).

**Table 1:** Protein identification by MALDI-TOF MS in leaves of tomato (*Solanum lycopersicum* L.) under pi starvation.

Spot No.	Protein Name	Plant species	gi number	Protein score	Regulation	Mr value	Theoretical *pI*	Experimental *pI*
	**Defense Response**							
7	Non-specific lipid-transfer protein 1	*Aegilops tauschii*	gi|475575069	60	**↑**	22318	4.5	5.0
8	probable WRKY transcription factor 13	*Populus euphratica*	gi|743837863	70	**↑**	26254	5.4	5.5
9	Non-specific lipid-transfer protein 1	*Aegilops tauschii*	gi|475575069	75	**↑**	22318	5.4	5.5
15	extra-large G protein 3, partial	*Arabidopsis lyrata*	gi|404359662	72	**↑**	19474	5.2	4.7
28	asymmetric leaves1 and rough sheath, putative	*Ricinus communis*	gi|255585187	75	**↑**	40007	4.5	4.7
33	extra-large G protein 3, partial	*Arabidopsis lyrata*	gi|404359802	89	**↑**	19346	4.2	4.9
	**Transcription/Translation**							
4	50S ribosomal protein L34	*Zea mays*	gi|226532888	64	**↓**	17803	6.1	6.3
5	pseudo-response regulator 7, partial	*Hordeum vulgare*	gi|566081769	68	**↓**	26306	6.4	6.4
6	zinc finger protein CONSTANS-LIKE 8	*Arabidopsis thaliana*	gi|79319580	66	**↓**	37500	6.9	7.0
14	pseudo-response regulator 7, partial	*Hordeum vulgare*	gi|566081765	74	**↓**	26405	5.4	4.7
17	Myb-related protein P	*Zea mays*	gi|618857433	67	**↓**	41351	5.3	5.4
18	pseudo-response regulator 7, partial	*Hordeum vulgare*	gi|566081765	74	**↓**	26405	5.2	5.5
20	Os08g0196700	*Oryza sativa*	gi|115475181	87	**↓**	20752	5.0	5.5
23	PREDICTED: mediator of RNA polymerase II transcription subunit 19a-like	*Musa acuminata*	gi|695040453	79	**↓**	25915	7.2	6.2
24	RNA-binding protein 34	*Glycine soja*	gi|734417436	74	**↓**	38282	7.5	6.2
26	TCP transcription factor	*Populus trichocarpa*	gi|429345853	76	**↓**	25946	4.0	4.4
27	PREDICTED: splicing factor U2af small subunit A isoform X2	*Camelina sativa*	gi|727435901	84	**↓**	29263	4.4	4.7
32	basic leucine zipper transcription factor	*Arabidopsis thaliana*	gi|21694632	68	**↓**	25611	3.5	4.8
43	pseudo-response regulator 7, partial	*Hordeum vulgare*	gi|570551886	79	**↓**	26255	5.4	5.1
44	basic leucine zipper transcription factor	*Arabidopsis thaliana*	gi|21694632	74	**↓**	25611	5.0	5.3
45	splicing factor PWI domain-containing protein	*Micromonas sp*.	gi|255071411	79	**↓**	28083	5.9	5.3
	**Carbohydrate and energy metabolism**							
2	BnaC01g10060D	*Brassica napus*	gi|674894438	60	**↑**	5489	4.2	4.5
11	PREDICTED: spidroin-1-like	*Zea mays*	gi|670366647	70	**↑**	22460	6.6	7.0
12	PREDICTED: spidroin-1-like	*Zea mays*	gi|670366648	70	**↑**	22460	4.0	4.1
16	coiled-coil domain-containing protein 86-like	*Beta vulgaris*	gi|731314876	68	**↑**	21751	4.1	4.6
34	BnaA07g24620D	*Brassica napus*	gi|674925867	90	**↑**	26493	4.3	4.4
40	BnaA07g36590D	*Brassica napus*	gi|674873484	79	**↑**	28145	4.1	4.3
25	BnaC07g03290D	*Brassica napus*	gi|674920219	70	**↑**	21276	4.2	4.4
	**Protein synthesis**							
21	PREDICTED: protein MAK16 homolog	*Nicotiana tomentosiformis*	gi|697186427	72	**↑**	35868	4.0	4.5
22	PREDICTED: ribosomal RNA processing protein 36 homolog	*Sesamum indicum*	gi|747041268	86	**↑**	28949	4.3	4.8
37	putative GAR1 protein	*Arabidopsis thaliana*	gi|21536739	64	**↑**	20968	3.2	4.7
	**Hypothetical/unknown proteins**							
10	hypothetical protein OsJ_08677	*Oryza sativa*	gi|125583968	75	**↑**	21181	5.9	6.0
13	TPA: hypothetical protein ZEAMMB73_684502	*Zea mays*	gi|414872386	66	**↑**	21032	5.4	4.4
19	uncharacterized protein LOC104648243	*Solanum lycopersicum*	gi|723713955	70	**↑**	21478	5.9	6.0
30	uncharacterized protein LOC100276640	*Zea mays*	gi|226500994	81	**↑**	27236	5.5	4.4
35	PREDICTED: uncharacterized protein LOC103492272	*Cucumis melo*	gi|659099823	77	**↑**	16965	4.1	4.4
39	predicted protein	*Physcomitrella patens*	gi|168027950	86	**↑**	23832	4.5	4.6
41	hypothetical protein M569_03306	*Genlisea aurea*	gi|527205647	63	**↑**	22499	5.9	4.3
42	hypothetical protein MIMGU_mgv1a014789mg	*Erythranthe guttata*	gi|604303492	75	**↑**	20368	6.7	4.1
	**Photosynthesis**							
3	mitochondrial-like, partial	*Camelina sativa*	gi|727483907	56	**↑**	89093	6.0	6.2
36	putative cyclin-L2	*Triticum urartu*	gi|210063837	71	**↑**	20749	4.2	4.6
46	ribosomal protein S9 (chloroplast)	*Acutodesmus obliquus*	gi|108773099	75	**↑**	16417	4.9	5.3
	**Other processes**							
1	mitochondrial carnitine/acylcarnitine carrier-like protein	*Cucumis melo*	gi|659132010	52	**↑**	30828	5.3	4.5
31	PREDICTED: nucleolin-like	*Brachypodium distachyon*	gi|357148214	70	**↑**	18327	5.6	4.2
38	Histone H1	*Aegilops tauschii*	gi|475378285	74	**↑**	19065	4.4	4.5
29	molybdopterin synthase catalytic subunit-like	*Fragaria vesca*	gi|470103798	79	**↑**	22552	5.4	4.4

**Note:** Arrows with up or down directions indicate up-regulation or down-regulation of proteins

The majority of protein spots were involved in transcription and translation processes and were either up or down-regulated in–Pi 2-DE gels. The protein spots involved in transcription and translation were identified as 50S ribosomal protein L34 (spot 4), Pseudo-response regulator 7, partial (spot 5, spot 14, spot 18 and spot 43), Zinc finger protein CONSTANS-LIKE 8 (spot 6), Myb-related protein P (spot 17), Os08g0196700 (spot 20), Protein MAK16 homolog (spot 21), Mediator of RNA polymerase II transcription subunit 19a-like (spot 23), RNA-binding protein 34 (spot 24), TCP transcription factor (spot 26), Splicing factor U2af small subunit A isoform X2 (spot 27 and spot 45), and Basic leucine zipper transcription factor (spot 32).

Furthermore, six up-regulated protein spots in–Pi 2-DE gels were involved in carbohydrate and energy metabolism and were identified as BnaC01g10060D (spot 2, spot 34, and spot 40), Spidroin-1-like (spot 11 and spot 12), and Coiled-coil domain-containing protein 86-like (spot 16). Similarly, three up-regulated protein spots involved in protein synthesis were identified as Protein MAK16 homolog (spot 21), Ribosomal RNA processing protein 36 homolog (spot 22), and Putative GAR1 protein (spot 37). In addition to important identified proteins described above some proteins were identified to be involved in other plant metabolic processes such as transport process, biosynthesis process, leaf development, DNA binding and few other protein spots were identified as hypothetical or unknown proteins.

Upon the assessment of each protein using the classifications as analyzed by gene ontology (www.geneontology.com), we classified 46 identified proteins into different functional groups (Fig 4B) under Pi starvation. The classification indicated that most of the identified proteins under Pi starvation were significantly related to stress/defense responses, photosynthesis, signal transduction or translational pathways, which alternately comprised an impression that tomato plants had the ability to respond to Pi starvation for homeostasis.

### Relative expression of *lePT1* and *lePT2* transcripts in tomato leaves to Pi starvation

**Fig 5:**
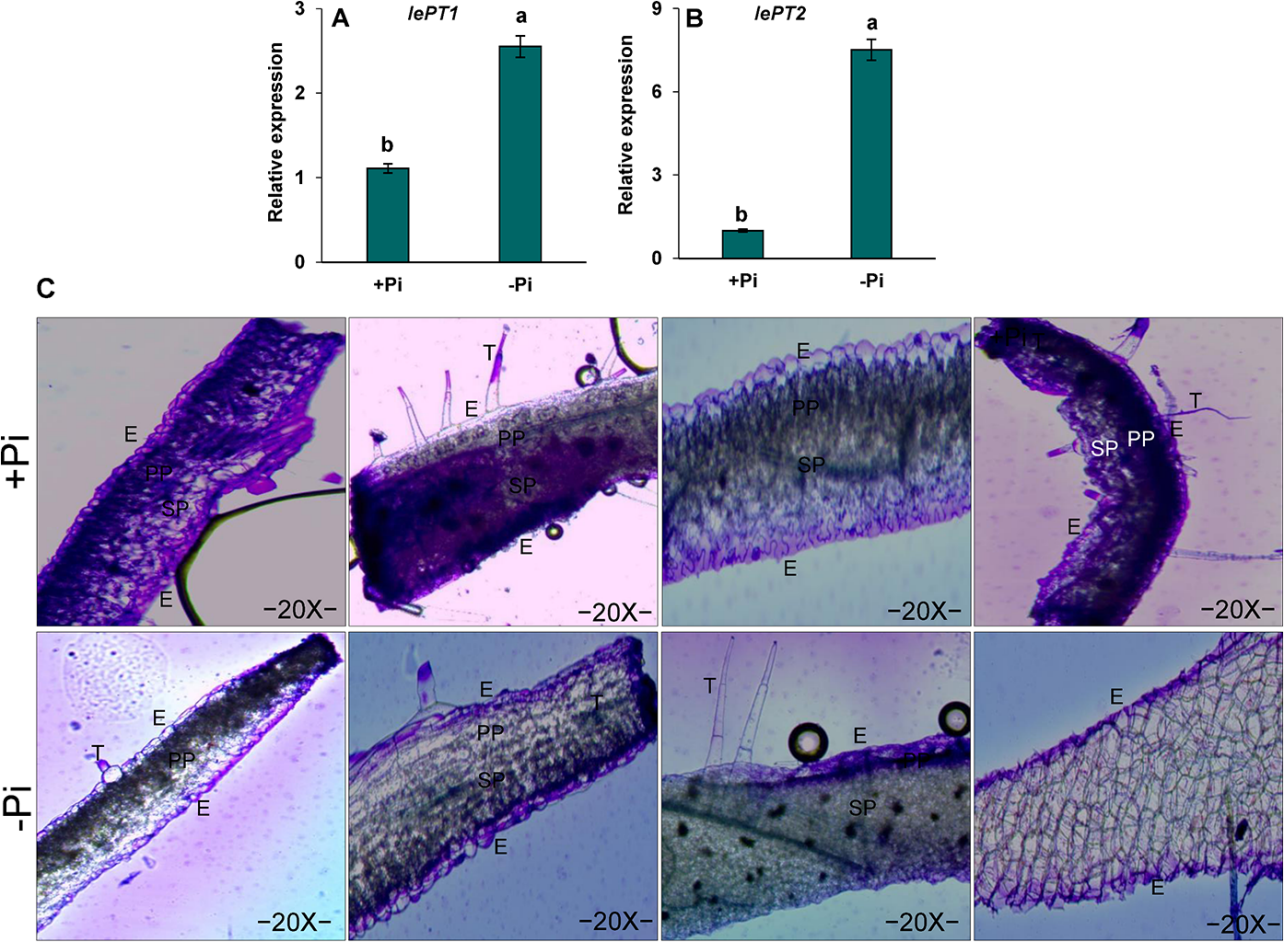
Relative expression and localization of phosphate transporters in leaves of tomato (*Solanum lycopersicum* L.) to Pi starvation. One week after germination tomato plants were supplied with sufficient Pi (1 M KH_2_PO_4_) or deficient Pi (0 M KH_2_PO_4_) for 10 days. (A) Relative expression of *lePT1* and *lePT2;* Vertical bars indicate Mean±SE of the means for n = 3. Means denoted by the different letter are significantly different at P≤0.05 according to the Tukey’s studentized range test (B) Localization of *lePT1* and *lePT2* observed under light microscope. E symbolizes epidermis, PP symbolizes palisade parenchyma, SP symbolizes sponge parenchyma, and T symbolizes trichome.

The relative expression of Pi transporter gene (*lePT1*) in tomato leaves was expressed significantly in higher amounts to 2.5 folds under Pi starvation (Fig 5A) compared to Pi-sufficient leaves. Similarly, the relative expression of *lePT2* gene significantly increased to 6 folds under Pi starvation (Fig 5B) compared to Pi-sufficient leaves. The increased expressions of *lePT1* and *lePT2* suggested a correlation between the amounts of phosphorus present in the medium in which tomato plants were grown.

### *lePT1* and *lePT2* localizations in tomato leaves to Pi starvation

*In situ* localization of *lePT1* and *lePT2* transcripts were performed to obtain information about tissue-specific expression of the phosphate transporters in leaves (Fig 5B). The signals of *lePT1* and *lePT2* transcripts were observed in the epidermis and palisade parenchyma. The presence of *lePT1* and *lePT2* was highly observed in the epidermis and palisade parenchyma in Pi-sufficient leaves. However, the *lePT1* and *lePT2* transcripts were widely observed in epidermis than palisade parenchyma in Pi-starved leaves.

## Discussion

### Physiological indices

Pi starvation is a major abiotic stress that limits crop productivity by 30–40% of the world’s arable land [27–28]. Moreover, to areas of low absolute soil P-content, Pi starvation arises in a soil where P is strongly bound to soil particles and has poor mobility to be absorbed by plants. The present study was conducted to determine the possible ways in tomato plants respond to Pi starvation and the tomato plants’ homeostasis at a proteome level. We observed chlorosis in leaves as a first symptom to Pi starvation (Fig 1A). The Pi starvation also led to decline in P content and photosynthetic pigments (total chlorophyll and carotenoid content) (Fig 1C and 1D). The reduction in photosynthetic pigments under Pi starvation has been reported in both in C_3_ [29–30] and C_4_ plants [31]. Pi starvation might result in a smaller size of stomatal opening, leading to the closure of stomata [32] which led to result in a decrease in photosynthetic pigments [10]. Another possible effect of Pi starvation in tomato plants was the production of reactive oxygen species which were observed by H_2_O_2_ and O_2_^-1^ localizations in our studies (Fig 2). The H_2_O_2_ and O_2_^-1^ are common stress indicators observed under various abiotic stresses in several plants including tomatoes [33]. The increase in ROS production might play an important role in modulating the induction of phosphate transporters expressed in response to Pi starvation [34–35].

### Proteomic analysis

Pi starvation affects many processes of the plants such as plant growth and development [36]. To attain novel insights in phosphorus homeostasis subjected to Pi starvation in tomato plants, we use two-dimensional gel electrophoresis combined with MALDI-TOF MS. For the relative protein profile by two-dimensional gel electrophoresis in leaves of tomato subjected to Pi starvation (Fig 3B) 46 protein spots were differentially expressed between +Pi and–Pi treatments. We observed that most of the protein spots in–Pi were up-regulated compared to +Pi treatments. The up-regulation of protein spots in–Pi tomato plants depicted the remobilization of Pi. Remobilization and acquisition are an important process for improving plant Pi-utilization efficiency and the maintenance of Pi homeostasis [37–38]. To obtain a deeper insight into the nature of Pi homeostasis and its acquisition, we assessed in further detail a number of the notable and differentially regulated proteins.

### Proteins related to photosynthesis

The abundance of proteins involved in photosynthesis was strongly affected by Pi starvation. The abundance of many photosynthetic proteins were up-regulated under Pi starvation (Fig 3B, Table 1). In particular, plant physiologists believe that Pi starvation has several impacts on photosynthesis such as influencing energy transfer across thylakoidal membranes [29], inactivating several pivotal enzymes involved in the Calvin cycle [31], and causing feedback inhibition of photosynthesis across thylakoid membrane through a reduction in electron transfer [39]. The expression of certain genes encoding proteins of photosynthesis, including photosystem I (PSI), photosystem II (PSII), ribulsoe-1,5-bisphophate oxygenase (RuBisCO), and chlorophyll a/b-binding proteins were repressed by Pi starvation [7–8, 40] whereas, our study showed the induction photosynthetic proteins. A possible variation in our study compared to previous study could be due several reason. The first reason might be because Pi starvation in previous studies was given for shorter period of time (7 days) such as in Arabidopsis [8] and (0 days) potato [40]. The other probable reason might be the genotype tolerance to Pi starvation used in previous studies compared to our studies. The up-regulation of photosynthetic proteins in our results suggested a promotion of the photosynthesis in tomato leaves even under Pi starvation. This showed that tomato plants resisted the Pi starvation up to a certain limit owing to Pi acquisitions and homeostasis predominantly due to up-regulation and localizations of Pi transporters in palisade parenchyma.

### Proteins related to defense response

Plants have developed defense response mechanisms to biotic and abiotic stresses, including Pi starvation [41–42]. The defense responsive proteins play a dual role in generating hydrogen peroxide in plants and function in signaling under stress conditions [43]. Here, we identified the number of protein spots (Table 1) under Pi starvation which belonged to defense mechanisms. It is well-known that plants have oxygen mechanism systems consisting of multiple enzymes to modulate the levels of reactive oxygen species (ROS) [43–44] and other isoenzymes with antioxidant activity [45]. The up-regulation of protein spots classified as defense response in tomato plants under Pi starvation suggested that these proteins might be involved in the reduction of ROS and regulation of redox status to protect plants from cell damage due to the toxicity of ROS. The present results indicated that tomato plants constrained the homeostasis of Pi due to the activation of stress-responsive proteins owing to Pi starvation.

### Proteins related to protein synthesis

Protein synthesis plays an important role in plant cells with regard to many physiological processes in response to unfavorable conditions [46–47]. The expression of an abundant number of protein spots under Pi starvation which plays a crucial role in protein synthesis was observed to be up-regulated under Pi starvation. The up-regulation of proteins related to protein synthesis has been previously reported in several plants under Pi starvation such as in *Arabidopsis thaliana* [48], *Oryza sativa* [49], and *Brassica napus* [11]. The up-regulation of proteins related to protein synthesis might enhance the translational process or enhance protein synthesis in tomato leaves under Pi starvation and maintain these proteins to encode the genes during acquisition and homeostasis under Pi starvation.

### Proteins related to transcription and translation

The modulation of protein expressions mainly involved in transcription and translation regulation is the key component for plants to survive under unfavorable environmental conditions [50–51]. Our protein identifications showed various numbers of proteins that had functional category of plant transcription and translation (Table 1). The 2-DE (Fig 3B) displayed proteins which were classified in translational category and mostly up-regulated under Pi starvation. A number of up-regulated transcription/translation proteins were identified in several plants under various abiotic stresses [52] while few reports indicated the down-regulation of transcription/translational proteins. Moreover, several studies also reported that proteins related to transcription and translation up-regulated under pi starvation such as in *Glycine max* [53] and *Brassica napus* [11]. The up-regulation of transcription and translation factor-related proteins in tomato plants portrayed a great acquisition and homeostasis under Pi starvation upon altering amino acids [54].

### Proteins related to carbohydrate and energy metabolism

The regulation of carbohydrate and energy metabolism related proteins resulted in up-regulation to Pi starvation (Fig 3B, Table 1). The up-regulation of these proteins might be due to the tolerance of the tomato cultivar to Pi starvation. The results indicated changes in carbohydrate and energy metabolism-related proteins under Pi starvation reduced in photosynthetic pigments as shown by our results of total chlorophyll and carotenoid contents (Fig 1C and 1D). The proteomic study performed on rice [41] and maize [55] seedlings also revealed the up-regulation of proteins related to carbohydrates or energy metabolism. The up-regulation of carbohydrate and energy metabolism-related proteins thus indicated alternate pathways of tomato plants for Pi homeostasis and its relative acquisition.

### Transcript levels and their localizations

The transcriptional analysis of phosphate transporters has been well-known in previous studies [5, 56–58]. A number of high affinity phosphate transporters are strongly expressed in roots, and are induced by phosphate starvation [59–60]. However, the transcription pattern of other high affinity phosphate transporters particularly *lePT1* and *lePT2* are transcribed both in leaves and roots of tomato [5]. In our studies, the gene expression levels of phosphate transporters (*lePT1* and *lePT2*) significantly increased in leaves under Pi starvation (Fig 5A and 5B). A similar observation has been reported in tomato leaves [5] and Arabidopsis roots [58]. Moreover, *in situ* localizations of *lePT1* and *lePT2* were observed in the epidermis and palisade parenchyma of Pi-sufficient than Pi-deficient leaves (Fig 5C) depicting a high coordination of plants to Pi-deficiency required for plant growth and photosynthesis. A tissue-specific localization of phosphate transporters such as high-affinity phosphate transporters has been reported in several other plants besides tomato such as chrysanthemum [57] and Arabidopsis [5, 58]. Our results thus, indicate that phosphate transporters constituted the ability of tomato to cope with Pi starvation.

## Conclusions

**Fig 6:**
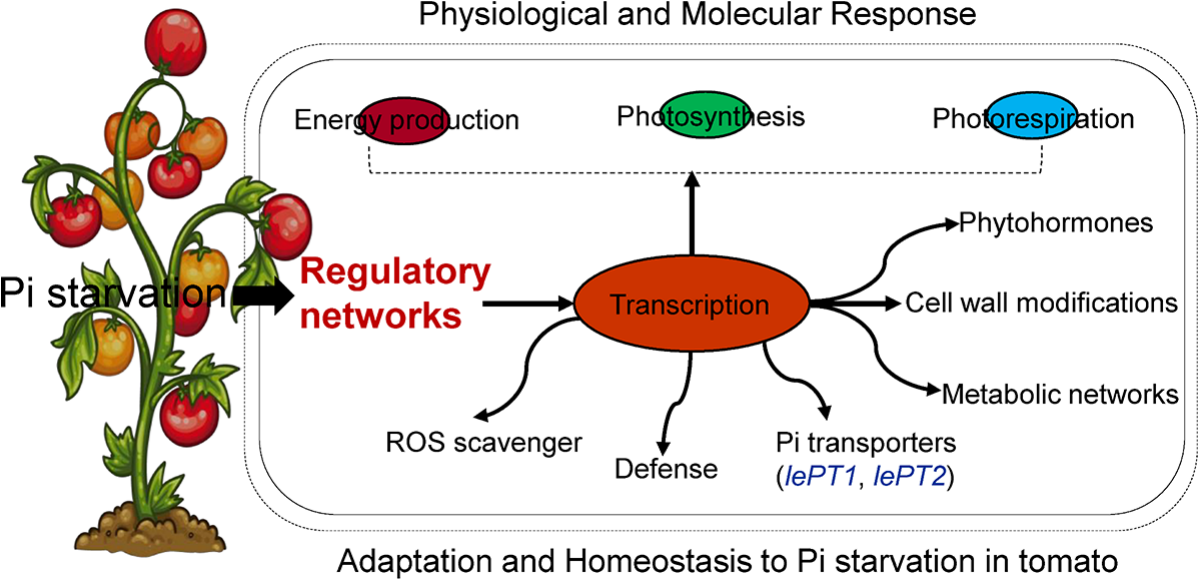
Schematic model and systematic pathway to Pi starvation, its tolerance and homeostatic mechanisms in tomato plants. Pi starvation affects the cellular processes like energy production, photosynthesis, photorespiration, and various metabolic pathways (transcription/translation) whereas, the regulatory pathways to defend Pi starvation for tolerance and its homeostasis were regulated in tomato plants as described in our proteome data and transcript levels.

Tomato is one of the highest consumed fruit throughout the world, one of the major challenge for its high quality and production is adaptive mechanisms of internal mineral deficiency. Among mineral deficiencies, Pi is naturally limiting in a soil due to a poor mobility. The major challenge in the present study was to explain Pi acquisition and its homeostasis at a proteome level. A number of proteins with various functions were identified. Some of the identified proteins were already known to be associated with abiotic stress but have not been detected in response to Pi starvation. The most significant proteins were identified as photosynthesis, defense responses, protein transcription/ translation related proteins. The identified proteins were mostly up-regulated in 2-DE gels which indicate tolerance or homeostasis of Pi under Pi starvation. Moreover, transcriptional regulation of Pi-transporter gene expressions and their localization regulated directly or indirectly to Pi acquisition which appeared to a very important part in homeostasis of Pi-starvation. A possible regulatory network for adaptation and homeostasis of Pi-starvation in tomato plants has been given diagrammatically in Fig 6 based on the results of our physiological, proteomic and transcriptional regulations.
